# High risk of drug-resistant tuberculosis in IGRA-negative contacts: should preventive treatment be considered?

**DOI:** 10.1007/s15010-024-02470-z

**Published:** 2025-01-21

**Authors:** Thomas Theo Brehm, Niklas Köhler, Hans-Peter Grobbel, Jürgen Welling, Anna Maria Mandalakas, Vinicius Fava, Erwin Schurr, Christoph Lange

**Affiliations:** 1 Department of Clinical Infectious Diseases, Research Center Borstel, Leibniz Lung Center, Parkallee 35, Borstel, Germany; 2 German Center for Infection Research (DZIF), Partner Site Hamburg-Lübeck-Borstel-Riems, Germany; 3 Division of Infectious Diseases, I. Department of Internal Medicine, University Medical Center Hamburg-Eppendorf, Hamburg, Germany; 4 Pneumologische Schwerpunktpraxis Lübeck, Lübeck, Germany; 5 Global Tuberculosis Program, Baylor College of Medicine and Texas Children’s Hospital, Houston, TX USA; 6 Program in Infectious Diseases and Immunity in Global Health, The Research Institute of the McGill University Health Centre, Montréal, Canada; 7 McGill International TB Centre, McGill University, Montréal, Canada; 8 Department of Human Genetics and Medicine, McGill University, Montréal, Canada; 9 Respiratory Medicine & International Health, University of Lübeck, Lübeck, Germany; 10 Institute for Infection Research and Vaccine Development (IIRVD), University Medical Center Hamburg-Eppendorf, Hamburg, Germany

**Keywords:** IGRA, Migrant, TPT, LTBI, MDR/RR-TB

## Abstract

**Purpose:**

Deciding whether to provide preventive treatment to contacts of individuals with multidrug-resistant (MDR) tuberculosis is complex.

**Methods:**

We present the diagnostic pathways, clinical course and outcome of tuberculosis treatment in eight siblings from a single family. Tuberculosis disease was diagnosed by *Mycobacterium tuberculosis* culture and molecular detection of *M. tuberculosis-specific* DNA from bronchopulmonary specimens using GeneXpert^®^ MTB/RIF. *M. tuberculosis* infection was diagnosed by an interferon-gamma release assay (IGRA; QuantiFERON^®^-TB Gold Plus). Whole exome sequencing for genetic predisposition to mycobacterial infection was performed in one patient.

**Results:**

Six of eight siblings aged 16–20 years from a migrant family of Somali origin were diagnosed with pulmonary MDR tuberculosis over a 12-month period. The remaining male siblings, aged 11 and 14 years, were asymptomatic during contact investigation. Chest radiographs, computed tomography (CT) scans, sputum cultures and nucleic acid amplification tests were negative, and the IGRA did not detect *M. tuberculosis* infection. A repeat CT scan eight months later was unremarkable, and repeated sputum cultures remained negative. In the absence of sufficient evidence of *M. tuberculosis* infection, no preventive treatment was offered. At month seven of consistent clinical observation, both children were diagnosed with pulmonary tuberculosis; the older with advanced disease and subsequent post-tuberculosis lung disease. Whole exome sequencing revealed no Mendelian variant associated with susceptibility to mycobacterial infection.

**Conclusion:**

When significant risk of tuberculosis transmission exists, close contacts of MDR tuberculosis patients should be offered preventive treatment with levofloxacin despite a negative IGRA test result.

**Supplementary Information:**

The online version contains supplementary material available at 10.1007/s15010-024-02470-z.

## Introduction

Tuberculosis remains a major global health concern with an estimated 10.8 million cases in 2023, including 400’000 cases of multidrug-resistant (MDR) and rifampicin-resistant (RR) tuberculosis [1]. In low-incidence countries, migrant communities bear a disproportionate burden of tuberculosis. Management of household contacts of tuberculosis patients requires a collaborative approach between clinicians and public health authorities that includes early diagnosis, contact tracing and screening, and initiation of tuberculosis preventive treatment. However, there is a gap in knowledge about how to manage individuals exposed to drug-resistant tuberculosis [2]. We present the unique case series of eight siblings from a migrant family who were all diagnosed with MDR- tuberculosis within 18 months in the absence of predisposing factors and address the challenges encountered in the clinical management of drug-resistant tuberculosis clusters.

## Case reports

**Fig. 1:**
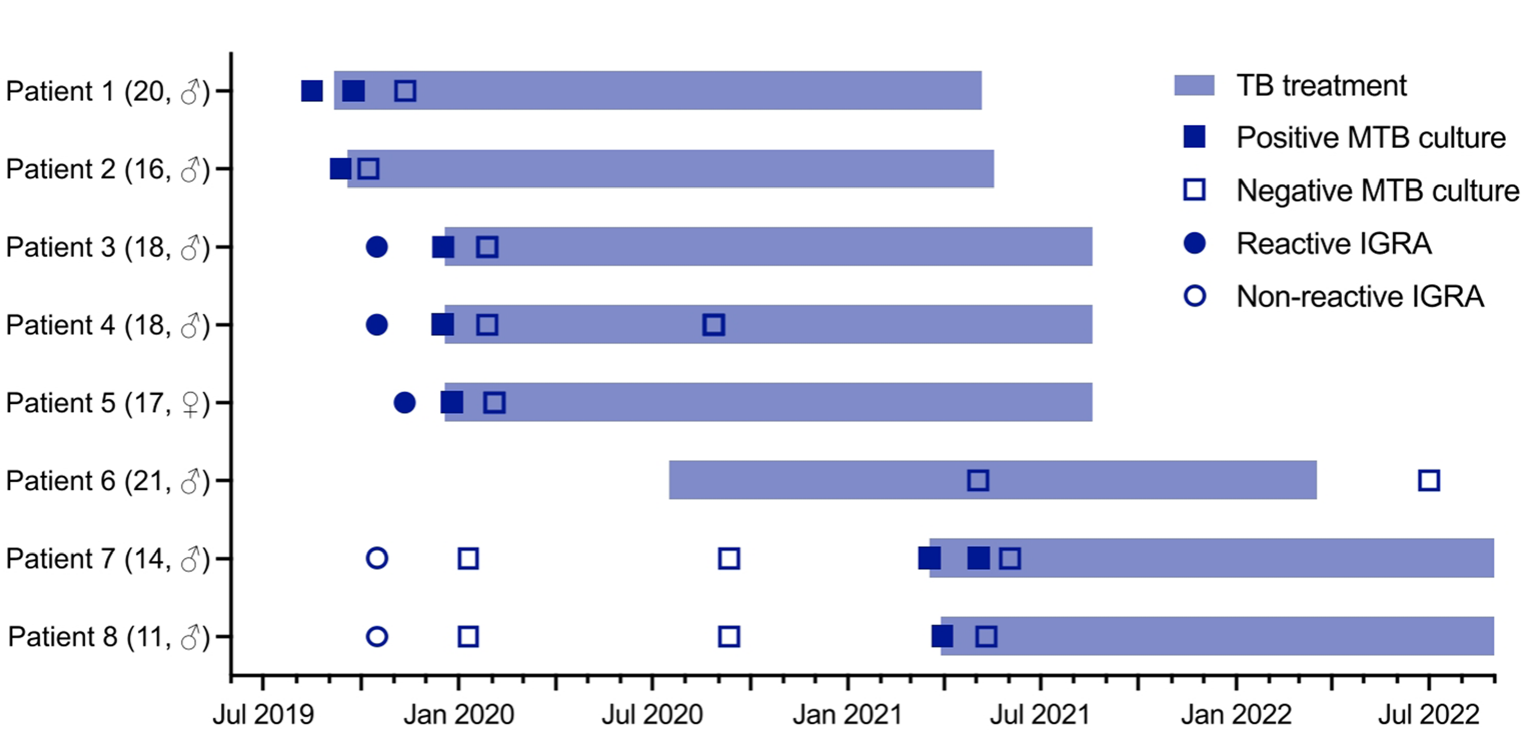
Treatment timeline of the eight siblings diagnosed with MDR tuberculosis. Legend: TB, tuberculosis; MTB, Mycobacterium tuberculosis; IGRA, interferon gamma release assay

**Fig. 2:**
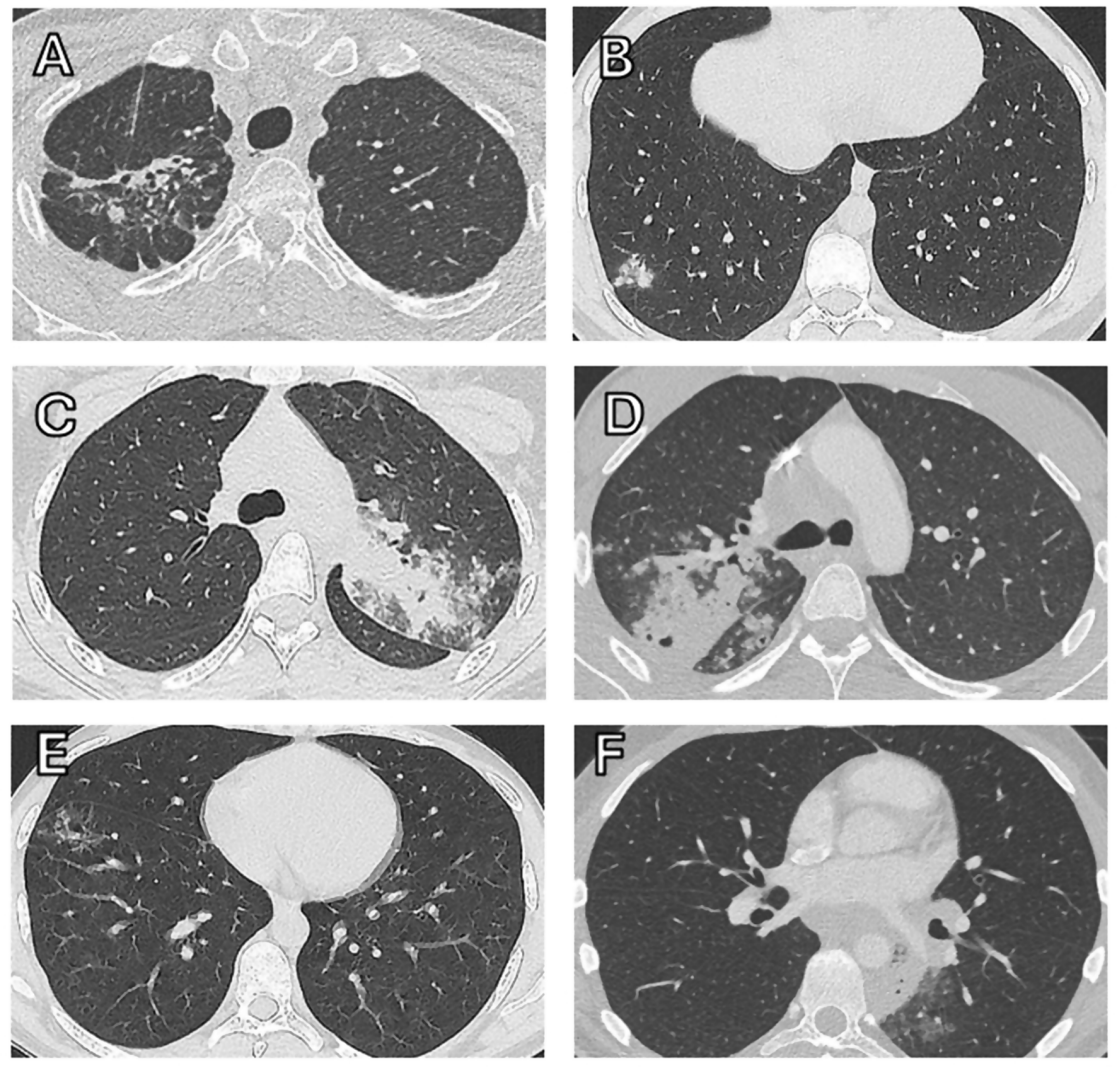
Chest CT scans of patients 1 to 6. Legend: **A**: Patient 1; **B**: Patient 2; **C**: Patient 3 **D**: Patient 4; **E**: Patient 5; **F**: Patient 6

**Fig. 3:**
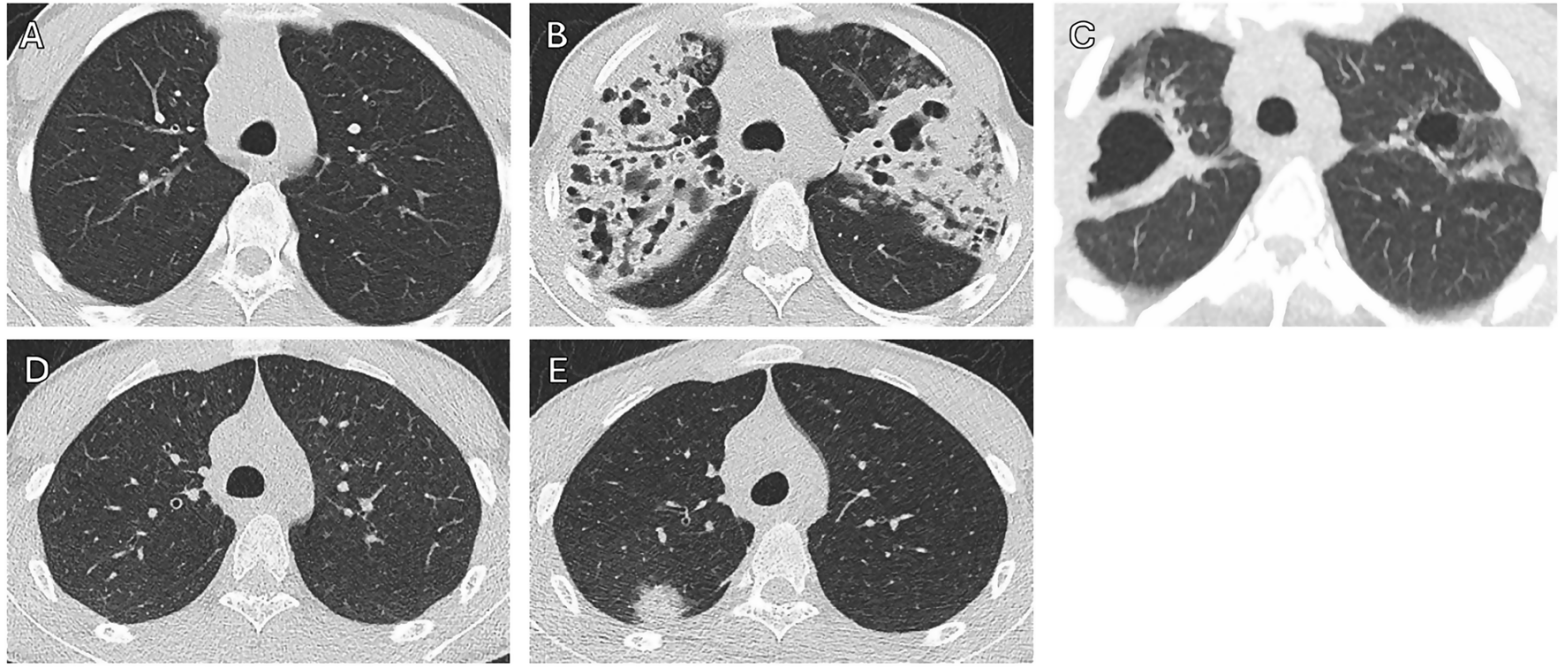
Serial chest CT scans of patients 7 and 8. Legend: **A**: Patient 7 in August 2020; **B**: Patient 7 in March 2021; **C**: Patient 7 in January 2024; **C**: Patient 8 in August 2020; **D**: Patient 8 in March 2021

In August 2019, a 20-year-old male patient (Patient 1) presented with a six-month history of chronic productive cough and an involuntary weight loss of 13 kg. He was born in Somalia and migrated to Syria in 2008, before settling in Germany in 2015. He had migrated together with his mother and seven siblings, all of whom lived together in one apartment. The father had passed away in Syria of an unknown respiratory illness. A chest radiograph and subsequent chest CT scan revealed infiltrates in the right lung (Fig. 1). Sputum samples showed acid-fast bacilli on smear microscopy and were positive for *Mycobacterium tuberculosis* by GeneXpert^®^ MTB/RIF (Cepheid, Sunnyvale, CA, USA) and mycobacterial culture. Genotypic drug susceptibility testing identified MDR tuberculosis with rpoB S531L, katG S315T1, and pncA K96T mutations, resulting in phenotypic resistance to rifampicin, isoniazid, and pyrazinamide. He was hospitalized and started treatment with levofloxacin, bedaquiline, linezolid, clofazimine, and terizidone. Over the next 12 months, five more of his siblings aged between 16 and 20 years (Patients 2–6), were diagnosed with pulmonary MDR tuberculosis (Figs. 1 and 2). As these cases occurred before the BPaLM (bedaquiline, pretomanid, linezolid and moxifloxacin) regimen was included in the World Health Organization (WHO) recommendations for MDR/RR tuberculosis in December 2022 [3], all patients were treated with individualized 18-month treatment regimens. The two youngest siblings, aged 14 and 11 years (Patients 7 and 8, respectively), were investigated as close contacts. They were found to be asymptomatic. Chest radiographs were unremarkable and QuantiFERON^®^-TB Gold Plus (QIAGEN, Germantown, MD, USA) tests were non-reactive in September 2019. This informed the decision not to initiate tuberculosis preventive treatment but inform on signs and symptoms of tuberculosis and to monitor them for signs and symptoms of tuberculosis as outpatients. Chest CT scans three and eleven months later showed no abnormalities, and induced sputum was negative by GeneXpert^®^ and mycobacterial culture. In March 2021, nineteen months after the diagnosis of tuberculosis in the first sibling, the general practitioner of the 14-year-old sibling (patient 7) reported the development of a cough and a weight loss of 10 kg. A subsequent chest CT revealed severe bilateral cavernous pulmonary infiltrates, and sputum samples now tested positive *for M. tuberculosis* with the same resistance profile as his siblings (Fig. 3). The still asymptomatic 11-year-old sibling (patient 8) simultaneously completed a chest CT that showed a right upper lobe infiltrate. He was also diagnosed with MDR tuberculosis disease with detectable *M. tuberculosis* DNA and documented growth of *M. tuberculosis* on culture from bronchoalveolar lavage but not from sputum. The mother had only non-specific changes on her chest CT scan and remained free of active disease. Both siblings started treatment with levofloxacin, bedaquiline, linezolid, clofazimine, and terizidone. *M. tuberculosis* culture of the 14-year-old converted on treatment after 18 days, and sputum cultures remained negative in the 11-year-old. Treatment was completed after 18 months. A follow-up CT scan revealed post-tuberculosis lung disease in the 14-year-old sibling with residual bipulmonary cavitary lesions. At the last follow-up in July 2024, 22 months after treatment completion, he had persistent restrictive lung disease with a forced expiratory volume in the first second (FEV_1_) of 3.32 l (71%) and a forced vital capacity (FVC) of 3.56 l (65%). All siblings tested negative for human immunodeficiency virus (HIV) and had normal lymphocyte counts. Given this rare clustering of tuberculosis cases within the family, whole exome sequencing for genetic predisposition to *M. tuberculosis* infection was performed in the 14-year-old (Patient 7), as he had the fastest progression and the most severe extent of disease. The detailed methods and results of whole genome sequencing are shown in Supplement 1 and 2. None of the identified variants were known to cause or had previously been associated with increased susceptibility to mycobacterial disease or infection. Four homozygous and 75 heterozygous rare protein-modifying variants were identified with a potential contribution to the risk of tuberculosis in this patient.

## Discussion

We present a unique case series of eight siblings from a family originating from a country with a high incidence of tuberculosis, all of whom lived in the same apartment and were diagnosed with MDR tuberculosis in Germany within an 18-month period, reminiscent of the catastrophic consequences for families affected by tuberculosis in previous centuries [4]. In two healthy contact siblings, tuberculosis disease and *M. tuberculosis* infection were initially excluded after intensive investigations. Given the negative IGRA results and the lack of recommendations for preventive therapies for contacts of index patients with drug-resistant tuberculosis without *M. tuberculosis* infection at that time, a “watch and wait” strategy was suggested. Both siblings subsequently developed tuberculosis.

The high rate of secondary MDR tuberculosis cases in this family is noteworthy. Household contacts of tuberculosis patients are at increased risk of infection and disease due to prolonged and intense exposure to index cases, with 90% of tuberculosis cases occurring within the first two years [5]. Meta-analyses have shown that between 30 and 47% of household contacts of MDR/RR tuberculosis patients have *M. tuberculosis* infection and that only between 3 and 8% develop active disease [5, 6]. There is no evidence that infection or disease rates in contacts differ between drug-resistant and drug-susceptible tuberculosis [7]. There were no predisposing factors for tuberculosis in any of the eight siblings, and whole exome sequencing failed to identify any variant known to be linked with known Mendelian or increased susceptibility to mycobacterial infection or disease. Hence, the precise factors contributing to the high rate of secondary tuberculosis cases remain elusive.

An important question arising from this case series is whether the administration of tuberculosis preventive treatment to the two youngest siblings, who initially had no evidence of *M. tuberculosis* infection or active disease could have prevented disease progression. According to the WHO recommendations, confirmation of *Mycobacterium tuberculosis* infection by tuberculin skin test (TST), tuberculosis antigen-based skin test (TBST) or IGRA is not required before starting TPT for MDR/RR-TB for child contacts and people with HIV or other immunocompromising conditions. In other populations, preventive treatment without confirmation of *M. tuberculosis* infection is not generally recommended, but the lack of test availability should not be a barrier to providing preventive treatment to those at risk of MDR/RR tuberculosis. The 2024 WHO Operational Handbook on Tuberculosis now recommends six months of daily levofloxacin monotherapy for contacts of people with MDR/RR tuberculosis [8]. This recommendation is based on evidence from the two randomised controlled trials CHAMP and V-QUIN, a systematic review of studies on the use of preventive treatment for MDR/RR tuberculosis and studies on the programmatic feasibility and acceptability of this regimen. While the TB-CHAMP [9] and V-QUIN trials [10] did not achieve statistical significance, a meta-analysis of these trials showed a 60% relative reduction in TB incidence among adult and child household MDR tuberculosis contacts [11]. In addition to these studies, the ongoing randomised prospective PHOENIx trial (NCT03568383) aims to evaluate the efficacy of tuberculosis preventive treatment with delamanid or isoniazid in preventing tuberculosis disease in high-risk household contacts of adults with MDR/RR- tuberculosis.

As the two youngest siblings tested negative on IGRA in September 2019, the clinicians decided to withhold tuberculosis preventive treatment and provide close outpatient follow-up. In retrospect, it can be argued that IGRA should have been repeated during follow-up to potentially detect early seroconversion and reassess the need for tuberculosis preventive treatment. Alternatively, tuberculosis preventive treatment should have been initiated despite the absence of a reactive IGRA due to the significant epidemiological risk. Importantly, the general sensitivity of IGRAs for the diagnosis of culture-proven active tuberculos isonly around 80% [12, 13]. Risk factors for negative IGRA resluts include immunodeficiency, young or advanced age, extrapulmonary tuberculosis, disseminated tuberculosis, concomitant tuberculosis treatment and smoking [14]. This underscores the importance of cautious interpretation of negative results in high-risk populations. Preventive treatment may have averted not only the development of severe pulmonary MDR tuberculosis but also the subsequent post-tuberculosis lung disease, which left one of the two children with residual bipulmonary cavitary lesions and persistent restrictive lung disease [15].

Delayed diagnosis of TB in migrants remains a significant challenge, often due to barriers such as limited access to health care, language difficulties, fear of stigma and unfamiliarity with local health systems. These delays can result in advanced disease progression by the time of diagnosis, as documented in our case series. Targeted public health interventions are needed to address this problem, including improved screening programmes, culturally sensitive health care approaches, and improved access to diagnostic services for migrant populations [16].

## Conclusion

This case series highlights the challenges and knowledge gaps in the clinical management of patients with drug-resistant tuberculosis and their contacts. Recent evidence suggests that six months of tuberculosis preventive treatment with levofloxacin is effective in preventing tuberculosis in contacts of fluoroquinolone-susceptible MDR/RR- tuberculosis patients with *M. tuberculosis* infection. The learning point from this case series is that tuberculosis preventive treatment should be strongly considered in healthy household contacts without confirmed *M. tuberculosis* infection when the epidemiological situation suggests a high risk of future development of active disease.

## Electronic supplementary material

Below is the link to the electronic supplementary material.

Supplementary Material 1

Supplementary Material 2

## Data Availability

No datasets were generated or analysed during the current study.

## References

[1] World Health Organization. Global tuberculosis report 2024. Geneva, World Health Organization; 2024.

[2] Gaskell KM, Allen R, Moore DAJ. Exposed! Management of MDR-TB household contacts in an evidence light era. Int J Infect Dis. 2019;80s:S13–6. 30825653 10.1016/j.ijid.2019.02.037

[3] WHO consolidated guidelines on tuberculosis. Module 4: treatment - drug-resistant tuberculosis treatment, 2022 update. Geneva: World Health Organization; 2022. 36630546

[4] Krejsa S. Sonder in Oldesloe: Eine Papiermüllerfamilie zwischen Courantthalern und Tuberculose. Dräger M, editor. Wachholtz, Neumuenster, Germany; 2009. ISBN ‎ 978-3529063589.

[5] Shah NS, Yuen CM, Heo M, Tolman AW, Becerra MC. Yield of contact investigations in households of patients with drug-resistant tuberculosis: systematic review and meta-analysis. Clin Infect Dis. 2014;58(3):381–91. 24065336 10.1093/cid/cit643 PMC3890332

[6] Fox GJ, Barry SE, Britton WJ, Marks GB. Contact investigation for tuberculosis: a systematic review and meta-analysis. Eur Respir J. 2013;41(1):140–56. 22936710 10.1183/09031936.00070812 PMC3533588

[7] Kodama C, Lange B, Olaru ID, Khan P, Lipman M, Seddon JA et al. Mycobacterium tuberculosis transmission from patients with drug-resistant compared to drug-susceptible TB: a systematic review and meta-analysis. Eur Respir J. 2017;50(4). 10.1183/13993003.01044-2017 29074544

[8] WHO operational handbook on tuberculosis. Module 1: prevention - tuberculosis preventive treatment, second edition. Geneva: World Health Organization; 2024. 39298638

[9] Hesseling AC, Purchase SE, Martinson NA, Fairlie L, Schaaf HS, Brigden J, et al. Levofloxacin Preventive Treatment in children exposed to MDR Tuberculosis. N Engl J Med. 2024;391(24):2315–26. 39693542 10.1056/NEJMoa2314318

[10] Fox GJ, Nhung NV, Cam Binh N, Hoa NB, Garden FL, Benedetti A, et al. Levofloxacin for the Prevention of Multidrug-resistant tuberculosis in Vietnam. N Engl J Med. 2024;391(24):2304–14. 39693541 10.1056/NEJMoa2314325

[11] Duong T, Brigden J, Simon Schaaf H, Garden F, Marais BJ, Anh Nguyen T, et al. A Meta-analysis of levofloxacin for contacts of Multidrug-resistant tuberculosis. NEJM Evid. 2025;4(1):EVIDoa2400190. 39693627 10.1056/EVIDoa2400190

[12] Sester M, Sotgiu G, Lange C, Giehl C, Girardi E, Migliori GB, et al. Interferon-γ release assays for the diagnosis of active tuberculosis: a systematic review and meta-analysis. Eur Respir J. 2011;37(1):100–11. 20847080 10.1183/09031936.00114810

[13] Diel R, Loddenkemper R, Nienhaus A. Evidence-based comparison of commercial interferon-gamma release assays for detecting active TB: a metaanalysis. Chest. 2010;137(4):952–68. 20022968 10.1378/chest.09-2350

[14] de Visser V, Sotgiu G, Lange C, Aabye MG, Bakker M, Bartalesi F, et al. False-negative interferon-γ release assay results in active tuberculosis: a TBNET study. Eur Respir J. 2015;45(1):279–83. 25359336 10.1183/09031936.00120214

[15] Ivanova O, Hoffmann VS, Lange C, Hoelscher M, Rachow A. Post-tuberculosis lung impairment: systematic review and meta-analysis of spirometry data from 14 621 people. Eur Respir Rev. 2023;32(168). 10.1183/16000617.0221-2022 PMC10113954 37076175

[16] Kunst H, Lange B, Hovardovska O, Bockey A, Zenner D, Andersen AB et al. Tuberculosis in adult migrants in Europe: a TBnet consensus statement. Eur Respir J. 2024. 10.1183/13993003.01612-2024 10.1183/13993003.01612-2024 PMC11883149 39672603

