# Supplementary material for: High risk of drug-resistant tuberculosis in IGRA-negative contacts: should preventive treatment be considered?
Source: Infection. 2025 Jan 21;53(4):1501–6. doi: 10.1007/s15010-024-02470-z (PMC12316788; doi:10.1007/s15010-024-02470-z)
Supplement: Supplementary file 1 — Supplementary Material 1 [file 15010_2024_2470_MOESM1_ESM.docx]

**Supplement 1**

**Methods of whole exome sequencing**

DNA extraction was conducted from blood samples. Coding sequences were enriched using the Nextera Flex for Enrichment Kit (Illumina) and the xGen Exome Research Panel v2 (IDT). Sequencing of the resulting libraries was performed on a NovaSeq6000 system (Illumina) using the 2x150 bp paired-end sequencing protocol. Subsequent optimization of the raw sequencing data was carried out utilizing FASTP v0.19.4. A whole exome sequence library was 100 base-pairs paired-end sequenced and then processed and aligned to the human genome followed by genomic variant detection and function annotation. Briefly, adaptors and low-quality reads were removed with TrimGalore v0.6.5 (1). High quality reads were then aligned to the human genome assembly hg38 with BWA MEM v0.7.17 using default parameters and duplicated reads were removed with PICARD v3.1.0 (2, 3). Next, variant calling was performed with HaplotypeCaller with GATK v4.2.6 (4). Genetic variants were annotated with wANNOVAR (5), for possible functional consequences. A total of 14,196 protein-altering variants were identified for the index case. We then proceeded to filter for those that had high predictive scores (CADD > 20) of impacting protein function. Next, we filtered for rare variants with minor allele frequency (MAF) > 1% in gnomAD v4.1 exome, a database of variant frequencies estimated on more than 730 thousand exomes. For the variants that passed the initial filtering, we used six different algorithms (SIFT, Polyphen2_HDIV, Polyphen2_HVAR, LRT, MutationTester and fathmm_MKL) to predict the deleterious effect of each mutation. We then separated mutation into two categories: (i) homozygote and (ii) heterozygote in the TB case. Homozygote variants predicted to be deleterious in 4/6 of the algorism and heterozygote variants predicted to be deleterious by all six algorithms are shown in **Supplemental Table 1**. We identified a total of 14,196 protein-altering variants in the index case. We then applied stepwise criteria to prioritize candidate variants via estimates of rarity and deleteriousness.

1. Martin M. Cutadapt Removes Adapter Sequences From High-Throughput Sequencing Reads. EMBnetjournal. 2011. <https://doi.org>: <https://doi.org/10.14806/ej.17.1.200>

2. Li H, Durbin R. Fast and accurate short read alignment with Burrows-Wheeler transform. Bioinformatics. 2009;25(14):1754-60. <https://doi.org:10.1093/bioinformatics/btp324>

3. Picard Toolkit. Broad Institute, GitHub repository. 2020.

4. Poplin R, Ruano-Rubio V, DePristo MA, Fennell TJ, Carneiro MO, Van der Auwera GA, et al. Scaling accurate genetic variant discovery to tens of thousands of samples. bioRxiv. 2018:201178. <https://doi.org:10.1101/201178>

5. Chang X, Wang K. wANNOVAR: annotating genetic variants for personal genomes via the web. J Med Genet. 2012;49(7):433-6. <https://doi.org:10.1136/jmedgenet-2012-100918>
